# Genome description of a potentially new species of Streptomyces isolated from the Indian Sundarbans mangrove

**DOI:** 10.1099/acmi.0.000892.v5

**Published:** 2024-12-16

**Authors:** Anwesha Ghosh, Simran Kaur Bhambra, Raghu Chandrasekaran, Punyasloke Bhadury

**Affiliations:** 1 Centre for Climate and Environmental Studies, Indian Institute of Science Education and Research Kolkata, Mohanpur 741246, West Bengal, India; 2 Integrative Taxonomy and Microbial Ecology Research Group, Department of Biological Sciences, Indian Institute of Science Education and Research Kolkata, Mohanpur 741246, West Bengal, India

**Keywords:** Bay of Bengal, estuary, mangrove, *Streptomyces*, Sundarbans

## Abstract

A potentially new species of *Streptomyces* was isolated from station 177 of the Sundarbans Seasonal Time Series in the Indian Sundarbans mangrove. The isolate was cultured from a sediment sample on TYS medium of salinity 15. Sequencing and annotation of the 16S rRNA showed 100% identity against *S. laurentii* NPS17 against GenBank/ENA/DDBJ. Annotation of the whole genome against the GTDB database showed closest identity with *S. terrae* SKN60 and belongs to the same clade as *S. roseicoloratus* TRM44457T and *S. laurentii* ATCC 31255. The genome is ~7.2 Mb and has a G+C% of 73%. The average amino acid identity was 85.01% with *S. roseicoloratus* and 80.34% with *S. roseolus*. The assembly reflected the presence of all essential genes and had 19 biosynthetic gene clusters predicted.

## Data Summary

The whole-genome sequence data reported in this manuscript are available in the NCBI as BioProject Accession Number PRJNA1141996. The BioSample Accession Number is SAMN42917027. All other data are included within this article or in the associated supplementary files.

## Announcement

*Streptomyces* is a filamentous, Gram-positive bacterial genus, belonging to the phylum Actinomycetota [1,2]. Around 850 species of *Streptomyces* are known till date. This genus has been reported from a variety of environments, including aquatic and terrestrial habitats, associated with plants as endophytes and as symbionts [3]. *Streptomyces* have large-sized genomes (usually between 8 and 10 Mb) with high G+C% (>70%) [4]. *Streptomyces* encode a myriad of natural products as they possess multiple biosynthetic gene clusters (BGCs; [5]). A *Streptomyces* species was isolated from a sediment sample of Station (Stn) number 177, representing the Sundarbans Seasonal Time Series (SuSTS) [Sundarbans Biosphere Reserve (SBR); 21° 39′ 14.4″N, 88° 33′ 24.47″E], which is part of the long-term ecological monitoring programme undertaken within the SBR of India Sundarbans. Surface sediment (~0–2 cm) sample was collected using a handheld corer (11 cm length and 2.5 cm diameter) from the intertidal zone of Stn177 during low tide when the sediment was exposed above water. The sediment sample was then scooped out and transferred into sterile collection bottles (100 ml total size) and transported to the lab under temperature-controlled conditions. In total 1 g weight wet sediment sample was grinded using a mortar and pestle to form fine grains, which were dried at 60 °C and then subdivided into ten portions, and each fraction was plated separately. The dried sediment was then plated on actinomycetes isolation agar (Cat. No. 17117; Merck, India) and incubated at 27 °C under aerobic conditions until colonies appeared on the plates. The first chalky colonies appeared on Day 7. Chalky colonies were selected based on ‘hyphae-like’ structures seen under bright-field microscopy by Gram staining. Branched, Gram-positive colonies were selected and re-streaked on AIA plates. Pure colonies were obtained with the first subculturing, but the isolate was re-streaked three consecutive times to ensure purity. The cells were isolated and grown in TYS medium (tryptone 3 g l^−1^, yeast extract 3 g l^−1^, sodium chloride 3 g l^−1^, dextrose 3 g l^−1^ and dipotassium hydrogen phosphate 1 g l^−1^) in estuarine water of salinity 15. The medium was supplemented with cycloheximide and nystatin (50 µg ml^−1^; SRL, India). Pure cultures were obtained by streaking one chalky white colony on TYS medium of salinity 15. The cells were subjected to Gram staining and subsequently observed under a bright-field microscope at 100× magnification (Bx53, Olympus, Japan) to ensure purity. Genomic DNA (gDNA) was extracted by using a modified published protocol [6]. Briefly, the cells were pelleted and lysis buffer (100 µl 50 mM Tris-HCl, 100 µl 20 mM EDTA, 100 µl 400 mM NaCl, 100 µl 750 mM sucrose and 10 µl 10% SDS; Merck) was added to the cells and incubated for 1 h at 50 °C. Then, 5 µl 10 mg ml^−1^ ProteinaseK (Amresco, USA) was added and the cells were incubated for 2 h at 55 °C. Following this, 10 µl 10 mg ml^−1^ of Lysozyme was added and cells were incubated for 2 h at 37 °C. Phenol:chloroform (Merck) was added in a ratio of 1 : 1 and the solution was mixed gently for 5 min and allowed to stand for 15 min. DNA was separated into the aqueous phase by centrifugation at 16 000 rcf for 12 min. The gDNA was precipitated by overnight incubation at −20 °C with 50 µl 3M CH_3_COONa (Merck, India) and 750 µl absolute ethanol (Merck, Germany). The gDNA was recovered by centrifugation at 16 000 rcf for 12 min and dissolving in 20 µl Tris-HCl. The gDNA was run on 1% agarose gel and quantified using a Nanodrop 2000c Spectrophotometer (Thermo Scientific, USA). The isolate was identified by amplifying the 16S rRNA using eubacterial primers (Fc27 and Rc1492) [7] and sequenced on ABI 3500 genetic analyzer based on Sanger sequencing chemistry. The generated chromatograms were manually checked using BioEdit 7.0. The forward and reverse sequences were joined to form a contig, and sequence identity was ascertained by blastn against GenBank.

The gDNA was quantified using Qubit, and the whole genome was sequenced on a MinION platform using Oxford Nanopore Technologies (ONT) sequencing chemistry. The genome library was prepared using a ligation sequencing kit (SQK-LSK109, ONT UK) using unfragmented DNA. No size-selective DNA fragmentation was performed. The library was purified using magnetic beads (Sergei Lab Supplies LLC, USA) and sequenced on R9.4.1 Flowcell. Basecalling was performed using Dorado 7.2.13 integrated in MinKNOW 23.11.4 using the hac model. The run generated 66 855 sequences with 247 909 981 bp and N50 of 7868 bp. The *de novo* genome assembly was filtered for long reads using flye (v2.9.3, [8]).

Software versions are stated, and all default parameters were used unless stated otherwise. The assembled genome quality was evaluated using QUAST (v5.2.0, [9]) and annotated using Prokka (v1.14.6) [10]. The genome map was created using Proksee [11]. The whole-genome sequence-based phylogeny was performed in the Type (Strain) Genome Server (TYGS) v391 (https://tygs.dsmz.de) [12]. The genome distances were estimated using the Genome blast Distance Phylogeny approach and the resulting intergenomic distances were used to calculate a balanced minimum evolution tree with branch support via FASTME 2.1.4, including SPR postprocessing [13]. Taxonomy classification, taxonomic novelty, presence of essential genes and average amino acid identity (AAI) were performed in MiGA Web 2.Orc2 (https://disc-genomics.uibk.ac.at/miga) [14]. Average nucleotide identity (ANI) was estimated in EZBioCloud version 20230823 (https://www.ezbiocloud.net/tools/ani) [15] against *S. roseicoloratus* (NCBI Assembly GCF_004117935.1). The taxonomic identity of the isolate was determined using the full-length amplified 16S rRNA sequence against GenBank/DDBJ/ENA and re-affirmed using the whole-genome data against GTDB. BGCs encoding natural products, also known as secondary metabolites, were identified using antiSMASH version 7.1.0 using relaxed detection strictness [16]. Similarity percentage stated with antiSMASH results shows the percentage of genes within the closest known compound that has a significant blast hit to genes within the current region.

**Fig. 1.**
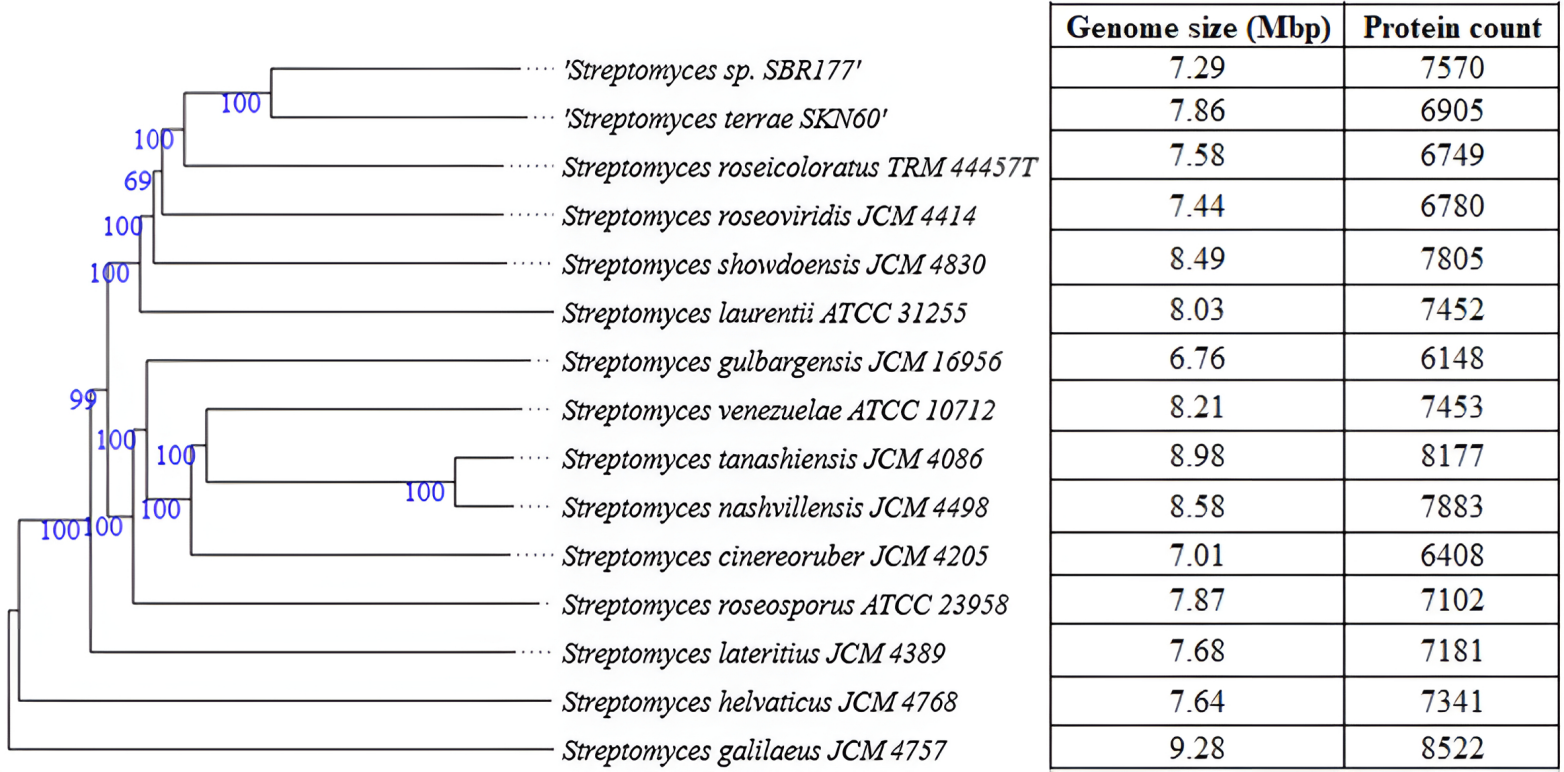
Phylogram showing phylogenetic relation of the isolate *Streptomyces* sp. SBR177 with other known *Streptomyces* spp. The numbers (in blue) represent the bootstrap value for each clade. The table on the right-hand side shows size of the genome and protein count of the closest identified relatives. All information has been obtained from the TYGS server.

**Fig. 2.**
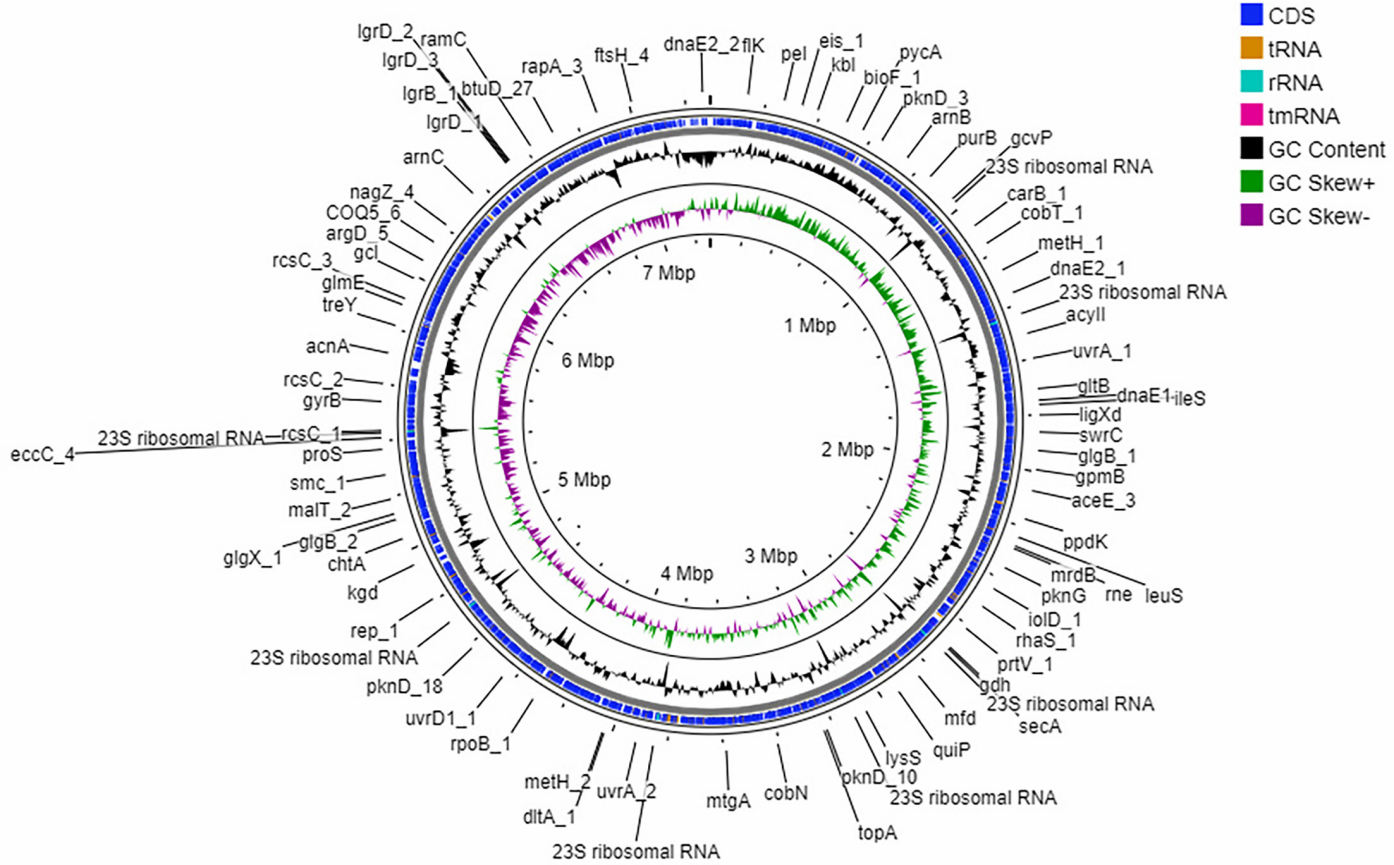
Genome map showing a circular map of the genome (representation only), identified genes, G+C content and GC Skew (+/) of *Streptomyces* sp. SBR177. The genome of *Streptomyces* sp. SBR177 is linear.

The isolate grows as chalky white colonies on TYS agar enriched with NaCl (Fig. S1, available in the online Supplementary Material). The colonies were hard to pick and had a distinct suffocative odour. Gram staining showed Gram-positive hyphae-like structures under 100× magnification (Fig. S1). The isolate was identified as a species of the genus *Streptomyces* with the closest identity with *S. roseicoloratus* and *S. laurentii* NPS17 (100% identity; accession number MZ645150MZ645150) using the full-length amplified 16S rRNA sequence (1545 bp). The whole-genome sequence showed the closest identity with *S. terrae* SKN60 against GTDB. The phylogenetic tree constructed in TYGS using the whole-genome data placed the isolate in the same clade as *S. roseicoloratus* TRM44457T and *S. laurentii* ATCC 31255 (Fig. 1). The isolate probably belongs to a new species that is not represented in Genome Taxonomy Database [https://gtdb.ecogenomic.org/]. The genome is about 7.2 Mb (7 290 923 bp) that assembled into one contig. The genome is linear and complete. Genome annotation indicated the presence of 7550 CDS, with 21 copies of rRNA genes, 87 copies of tRNA and 1 tmRNA (Fig. 2). The G+C content of the genome is about 73%. The average AAI was 85.01% with *S. roseicoloratus* (GCF 004117935 1^T^) and 80.34% with *S. roseolus* (GCF 014649855 1T). OrthoANI showed 86.97% identity against *S. roseicoloratus*.

The genome is predicted to contain complete central carbohydrate metabolism, including the pentose phosphate pathway and genes linked to the TCA cycle. Complete genetic pathways for galactose degradation, nucleotide sugar biosynthesis and UDP-*N*-acetyl-d-glucosamine biosynthesis were identified. The genome contains genes for formaldehyde assimilation, including *hlx*A (encoding for 3-hexulose-6-phosphate synthase), *hlx*B (6-phospho-3-hexuloisomerase), *pfk* and *pfp* (ATP-dependent phosphofructokinase/diphosphate-dependent phosphofructokinase) and *fba*A (fructose-bisphosphate aldolase). This implies that the isolate may use C1 substrate (i.e. methanol or format) and convert it into formaldehyde, which can then be assimilated through downstream reactions, including the ribulose monophosphate cycle, the xylulose monophosphate cycle or the serine cycle. Such cycles have been previously reported from methylotrophs [17]. The genes involved in the ribulose monophosphate cycle, as part of the methane metabolism, were identified in the genome. Genes encoding for enzymes involved in coenzyme F420 biosynthesis, a flavin-derived coenzyme involved in redox reactions in methanogens, were also present. These include *cof*C (2-phospho-l-lactate/phosphoenolpyruvate guanylyltransferase), *cof*D (2-phospho-l-lactate transferase), *cof*E (l-glutamate ligase), *cof*G (7,8-dimethyl-8-hydroyx-5-deazariboflavin synthase), *cof*H (5-amino-6-(d-ribitylamino) uracil-l-tyrosine 4-hydroxyphenyl transferase) and *fbi*B (dehydro coenzyme F420 reductase). The assimilatory sulphate reduction pathway genes were also identified, comprising *cys*N (sulphate adenylyltransferase subunit 1), *cys*C (adenylysulfate kinase), *cys*D (sulphate adenylyltransferase subunit 2), *cys*H (phosphoadenosine phosphosulfate reductase) and *sir* (sulphite reductase). This pathway reduces sulphate (oxidation state +6) to sulphide (oxidation state −2) [18]. Genes for C10-C20 isoprenoid biosynthesis, including *idi* (isopentenyl-diphosphate delta-isomerase) and *ids*A (geranylgeranyl diphosphate synthase type I), were identified in the genome. Genes encoding Type I polyketide structure, among other terpenoids and polyketides, were identified in the genome (Fig. S3). Notable among these are genes involved in the biosynthesis of ansamycins (a type of antibiotics). Four genes, including *asm*13 to *asm*16, were found. Among other secondary metabolites, the genome appears to encode for phenylpropanoid biosynthesis that can use phenylalanine, tyrosine and tryptophan to produce various forms of lignin, including *p*-hydroxyphenyl lignin, guaiacyl lignin, 5-hydroxy guaiacyl lignin and syringyl lignin. The isolate could biosynthesize penicillin owing to the presence of *cef*D gene that catalyse isopenicillin N into penicillin N and *pen*P as well as *pac* that can generate penicilloic acid and 6-aminopenicillanic acid, respectively. Bacterial type II PKS BGCs code for the biosynthesis of aromatic polyketides, including tetracycline (antibiotic) and doxorubicin (anti-tumour). Results from antiSMASH identified the PKS flaviolin BGC that can produce the compound flaviolin (Fig. S3). The desferrioxamine B BGC (NRPS-independent, lucA/lucC-like siderophores) coding for desferrioxamin B/desferrioxamine E also showed 100% similarity with known secondary metabolites [19]. A ribosomally synthesized and post-translationally modified thiopeptide encoding gene cluster was identified with 72% similarity to known genes. This codes for radamycin/globimycin type antimicrobial agents. The biosynthetic thiopeptide is YcaO. Other domains include the lantibiotic dehydratase domain, lantibiotic biosynthesis dehydratase and SAM-domain protein that made up the core biosynthetic genes. TetR family transcriptional regulation and drug resistance transporter (EmrB/QacA) were also present. The domain-producing ectoine, a compatible solute that prevents osmotic stress in saline environments [20], was also identified.

## supplementary material

10.1099/acmi.0.000892.v5 Uncited Supplementary Material 1.

## Abbreviations

AAI: amino acid identity
AIA: actinomycetes isolation agar
ANI: average nucleotide identity
BGCs: biosynthetic gene clusters
gDNA: genomic DNA
GTDB: genome taxonomy database
PKS: polyketide synthase
SAM: sequence alignment format
SBR: Sundarbans Biosphere Reserve
SPR: subtree pruning and regrafting
TYGS: type genome server
TYS: tryptone yeast and sucrose

## References

[1] Barka EA, Vatsa P, Sanchez L, Gaveau-Vaillant N, Jacquard C (2016). Taxonomy, physiology, and natural products of actinobacteria. Microbiol Mol Biol Rev.

[2] Oren A, Garrity GM (2021). Valid publication of the names of forty-two phyla of prokaryotes. Int J Syst Evol Microbiol.

[3] Alam K, Mazumder A, Sikdar S, Zhao Y-M, Hao J (2022). *Streptomyces*: the biofactory of secondary metabolites. Front Microbiol.

[4] Hopwood DA (2019). Highlights of Streptomyces genetics. *Heredity*.

[5] Ward AC, Allenby NE (2018). Genome mining for the search and discovery of bioactive compounds: the Streptomyces paradigm. FEMS microbiology letters.

[6] Boström KH, Simu K, Hagström Å, Riemann L (2004). Optimization of DNA extraction for quantitative marine bacterioplankton community analysis. Limnol Ocean Methods.

[7] Lane DJ, Stackebrandt E, Goodfellow M (1991). Nucleic Acid Techniques in Bacterial Systematics.

[8] Kolmogorov M, Yuan J, Lin Y, Pevzner PA (2019). Assembly of long, error-prone reads using repeat graphs. Nature biotechnology.

[9] Gurevich A, Saveliev V, Vyahhi N, Tesler G (2013). QUAST: quality assessment tool for genome assemblies. Bioinformatics.

[10] Seemann T (2014). Prokka: rapid prokaryotic genome annotation. Bioinformatics.

[11] Grant JR, Enns E, Marinier E, Mandal A, Herman EK (2023). Proksee: in-depth characterization and visualization of bacterial genomes. Nucleic Acids Res.

[12] Meier-Kolthoff JP, Göker M (2019). TYGS is an automated high-throughput platform for state-of-the-art genome-based taxonomy. Nat Commun.

[13] Lefort V, Desper R, Gascuel O (2015). FastME 2.0: A Comprehensive, Accurate, and Fast Distance-Based Phylogeny Inference Program. Molecular biology and evolution.

[14] Rodriguez-R LM, Gunturu S, Harvey WT, Rosselló-Mora R, Tiedje JM (2018). The Microbial Genomes Atlas (MiGA) webserver: taxonomic and gene diversity analysis of Archaea and Bacteria at the whole genome level. Nucleic Acids Res.

[15] Chalita M, Kim YO, Park S, Oh H-S, Cho JH (2024). EzBioCloud: a genome-driven database and platform for microbiome identification and discovery. Int J Syst Evol Microbiol.

[16] Blin K, Shaw S, Augustijn HE, Reitz ZL, Biermann F (2023). antiSMASH 7.0: new and improved predictions for detection, regulation, chemical structures and visualisation. Nucleic Acids Res.

[17] Reiter MA, Bradley T, Büchel LA, Keller P, Hegedis E (2024). A synthetic methylotrophic *Escherichia coli* as A chassis for bioproduction from methanol. Nature catalysis.

[18] Kushkevych I, Cejnar J, Treml J, Dordević D, Kollar P (2020). Recent advances in metabolic pathways of sulfate reduction in intestinal bacteria. Cells.

[19] Giddings L-A, Lountos GT, Kim KW, Brockley M, Needle D (2021). Characterization of a broadly specific cadaverine N-hydroxylase involved in desferrioxamine B biosynthesis in *Streptomyces sviceus*. PLoS One.

[20] Czech L, Hermann L, Stöveken N, Richter AA, Höppner A (2018). Role of the extremolytes ectoine and hydroxyectoine as stress protectants and nutrients: genetics, phylogenomics, biochemistry, and structural analysis. Genes.

