# Supplementary material for: Genome description of a potentially new species of Streptomyces isolated from the Indian Sundarbans mangrove
Source: Access Microbiol. 2024 Dec 16;6(12):000892.v5. doi: 10.1099/acmi.0.000892.v5 (PMC11648730; doi:10.1099/acmi.0.000892.v5)
Supplement: Uncited Supplementary Material 1. [file acmi-6-00892-s001.pdf]

Supplementary Information

Fig. S1: The left-hand panels show the filaments observed under 100X magnification of bright-field microscope. The right-hand panel showed the colony morphology as seen from the front and back of the TYS agar plate.

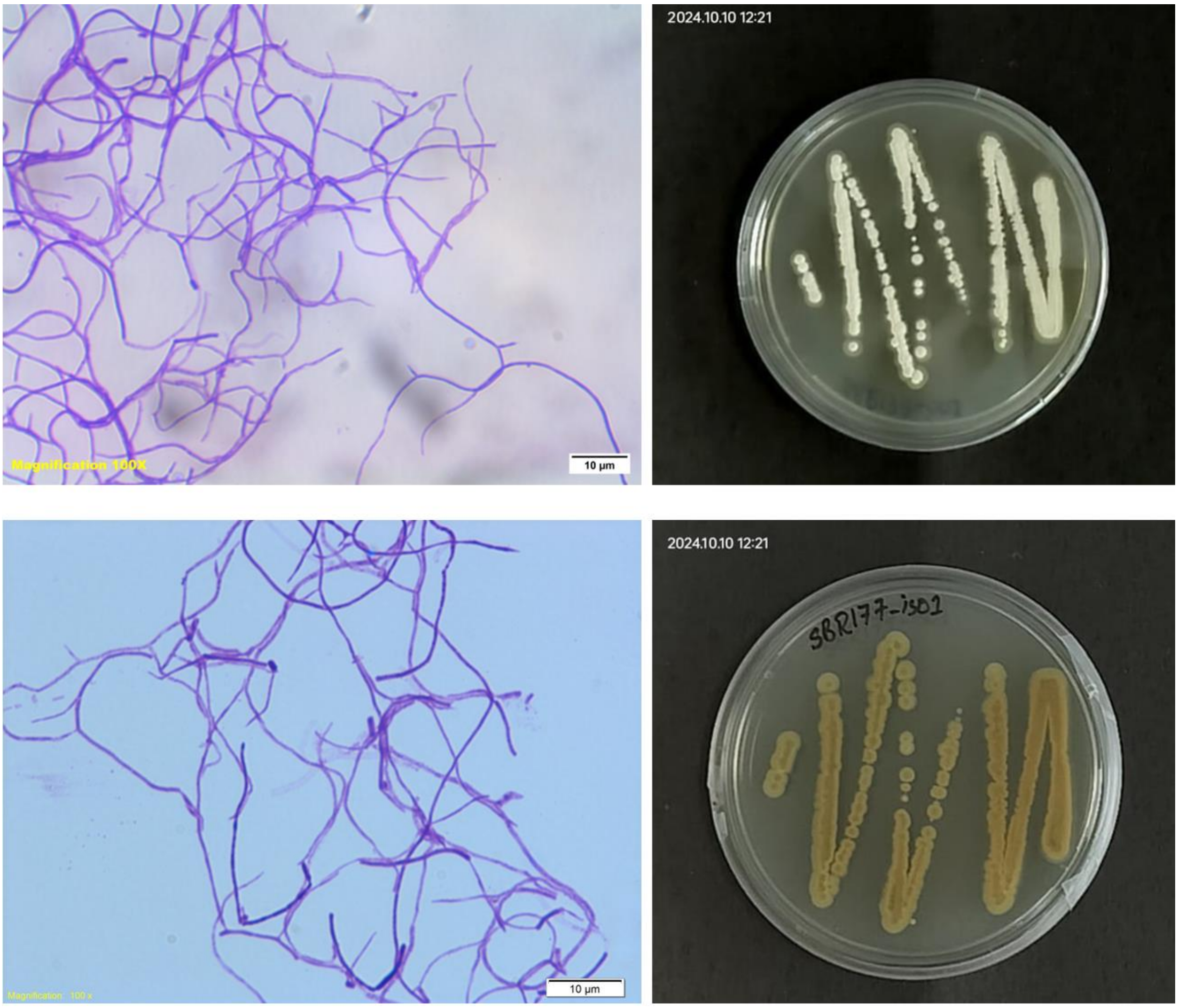

Fig. S2: Phylogenetic tree constructed from 16S rRNA sequence obtained from the genome. The numbers (in blue) show the bootstrap value of the node. The black bars show size of genomes of the closest identified relatives of *Streptomyces* sp. SBR177. The brown bars show proteins and the blue bars show the size of the 16S rRNA. The shown tree has been obtained from TYGS output.

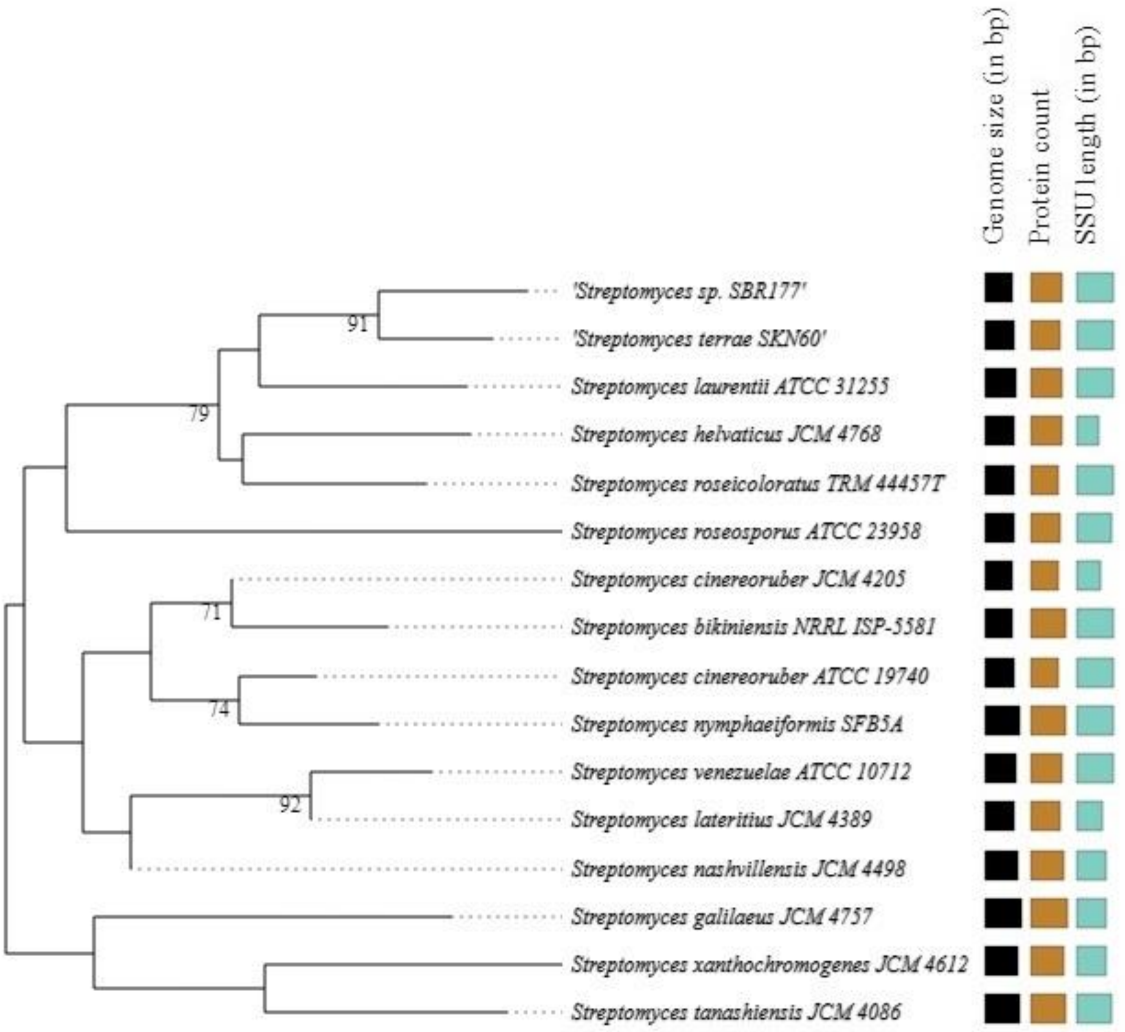

Fig. S3: Summary of results of AntiSMASH showing the identified NRPS and PKS genes from the genome assembly of *Streptomyces* sp. SBR177.

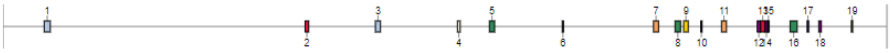

Region	Type	From	To	Most similar known cluster		Similarity
Region 1	terpene, T3PKS	340,079	390,562	flaviolin/1,3,6,8-tetrahydroxynaphthalene	Polyketide	100%
Region 2	NI-siderophore	2,492,278	2,522,062	desferrioxamin B/desferrioxamine E	Other	100%
Region 3	betalactone, T1PKS	3,072,078	3,114,094	A-201A	Other	6%
Region 4	nucleoside	3,748,414	3,771,524	TVA-YJ-2	RiPP	7%
Region 5	NRPS-like	4,012,341	4,055,628	xantholipin	Polyketide	10%
Region 6	melanin	4,613,421	4,623,831	istamycin	Saccharide	8%
Region 7	T3PKS	5,366,293	5,407,426	flaviolin/1,3,6,8-tetrahydroxynaphthalene	Polyketide	66%
Region 8	NRPS	5,543,692	5,588,910	auroramycin	Polyketide	17%
Region 9	thiopeptide	5,614,966	5,652,136	radamycin/globimycin	RiPP	72%
Region 10	RiPP-like	5,755,810	5,766,286			
Region 11	PKS-like	5,926,642	5,967,634	tyrobetaine	NRP	53%
Region 12	terpene	6,221,397	6,248,003	hopene	Terpene	69%
Region 13	NI-siderophore	6,250,608	6,283,191	peucechelin	NRP	30%
Region 14	terpene	6,287,323	6,308,213	ebelactone	Polyketide	5%
Region 15	RiPP-like	6,308,238	6,319,062			
Region 16	NRPS	6,492,727	6,550,813	desotamide	NRP	31%
Region 17	hydrogen-cyanide	6,633,082	6,645,443	aborycin	RiPP	14%
Region 18	terpene	6,728,885	6,749,952	bleomycin A2/bleomycin B2	NRP+Polyketide+Saccharide	4%
Region 19	ectoine	6,997,944	7,008,354	ectoine	Other	100%
